# Veasey’s Ophthalmology

**Published:** 1903-10

**Authors:** 


Veasey's Ophthalmology.—A Manual of Diseases of the Eye. For Students and General Practitioners. By Clarence A. Veasey, A. M., M. D., Demonstrator of Ophthalmology in Jefferson Medical College, Philadelphia. 12mo, 410 pages, with 194 engravings and 10 full-page colored plates. Cloth, $2.00 net. Lea Brothers & Co., Publishers, Philadelphia and New York.

This is a conveniently arranged, well written, and authoritative work on practical ophthalmology, and is likely to prove of more value even to specialists than are most of the larger and more pretentious works on the subject. The author has wisely avoided lengthy and profitless discussions on unsettled theories, thus saving space for a consideration of matters of practical importance to men engaged in actual practice. R. & L.

